# Disentangling mature cow weight and body condition score: a comparative single-step GWAS of phenotypic pre-adjustment vs. recursive modeling

**DOI:** 10.1093/jas/skag231

**Published:** 2026-07-28

**Authors:** Ayooluwa O Ojo, Henrique A Mulim-McCarthy, André Garcia, Kelli Retallick-Riley, Hinayah R Oliveira

**Affiliations:** Department of Animal Sciences, Purdue University, West Lafayette, IN 47907, United States; Department of Animal Sciences, Purdue University, West Lafayette, IN 47907, United States; Angus Genetics Inc., American Angus Association, St Joseph, MO 64506, United States; Angus Genetics Inc., American Angus Association, St Joseph, MO 64506, United States; Department of Animal Sciences, Purdue University, West Lafayette, IN 47907, United States

**Keywords:** functional analyses, genetically independent, metabolism, pathways, modeling approaches

## Abstract

Mature cow weight (MWT) is a trait genetically correlated with body condition score (BCS). Previous research has shown that sire rankings can shift depending on how BCS is accounted for, indicating that different modeling strategies can influence selection outcomes. The recursive modeling approach has been established as a method for obtaining MWT that is genetically independent of BCS, providing an alternative to phenotypic pre-adjustment. The objective of this study was to determine whether different modeling approaches capture different genetic architectures or merely produce statistical artifacts. Genome-wide association studies (GWAS) and functional genomic analyses were performed to compare the genomic architecture of phenotypically pre-adjusted MWT (MWT_adj_) with MWT that is genetically independent of BCS, obtained using the recursive approach (MWT_RM_). A total of 42 significant SNP across 8 chromosomes were identified for MWT_adj_ and 44 SNP across nine chromosomes for MWT_RM_, with 28 SNP shared between models. These variants corresponded to 107 annotated genes in MWT_adj_ and 137 in MWT_RM_, including 62 shared genes. Major association signals were concentrated on BTA20, BTA7, and BTA14 for both models, with all significant SNP jointly explaining 3.93% of the total additive genetic variance for MWT_adj_ and 4.29% for MWT_RM_. The Pearson correlation coefficient of estimated SNP effects between the models was 0.76, while the correlation of genomic estimated breeding values was 0.87. Compared to MWT_adj_, which shared 19 genes with unadjusted MWT, the MWT_RM_ shared 33 genes. In addition, 28 of the 31 pathways identified for MWT_RM_ were also identified for unadjusted MWT, whereas no Gene Ontology pathways were shared between MWT_adj_ and unadjusted MWT. The MWT_RM_ was associated with genes annotated to growth, skeletal development, feed efficiency, and carcass-related traits, whereas MWT_adj_ identified a distinct set of genes and pathways. Despite these differences, MWT_adj_ and MWT_RM_ converged on similar core biological signals, highlighting that they effectively capture the primary genetic drivers of MWT independent of BCS.

## Teaser

Phenotypic pre-adjustment of mature cow weight for body condition score and recursive modeling to obtain MWT genetically independent of BCS converged on largely overlapping biological pathways, indicating consistent core genetic drivers across modeling approaches.

## Introduction

Body condition score (BCS) is an informative indicator of a beef cow’s nutritional status and long-term productivity (D’Occhio et al. 2019; Pulina et al. 2021). As a direct measure of energy reserves, BCS influences key biological processes, including reproduction (Cooke et al. 2021; Carvalho et al. 2022), lactation (Briggs et al. 2022; Orquera-Arguero et al. 2023), and maintenance efficiency (Delver et al. 2023), thereby playing a pivotal role in the economic performance of beef cattle production systems. Because BCS also affects the weight at which cows express their mature size (Snelling et al. 2019), genetic evaluations and selection programs for mature cow weight (MWT) should account for differences in body condition. For instance, in the American Angus Association (AAA) population, BCS accounts for approximately 16% of the variation in cow weight (Northcutt et al. 1992). Northcutt et al. (1992) emphasized that evaluations of mature cow size should explicitly adjust for the degree of fatness expressed at a given weight. The authors also recommended incorporating routinely recorded BCS information, collected alongside weight and height data, as adjustment factors into the genetic evaluations for MWT. Consequently, AAA adopted BCS-based adjustment factors to reduce bias in MWT breeding values, recognizing that unadjusted records affect sire rankings and obscure true genetic differences in MWT (Northcutt et al. 1992; Northcutt and Wilson 1993).

However, BCS itself is a heritable trait that is also genetically correlated with MWT (Ribeiro et al. 2022; Ojo et al. 2025a). In American Angus cattle, BCS showed a heritability of 0.18 and a positive genetic correlation with MWT (r_g_=0.57), demonstrating that a substantial portion of their association reflects shared genetic architecture rather than purely environmental effects (Ojo et al. 2025a). Because part of the relationship between BCS and MWT arises from underlying genetic covariance, not just environmental influences, phenotypic pre-adjustment alone may fail to capture the true genetic merit of mature size in cows, and it might not fully disentangle their shared biological and genetic basis (Aschard et al. 2015; Ojo et al. 2025b). Phenotypic pre-adjustment removes the observed association between BCS and MWT by adjusting the MWT phenotype for BCS before genetic evaluation. As a result, both environmental and genetically mediated pathways linking BCS and MWT are removed from the phenotype. To address this limitation, alternative modeling strategies, such as the recursive modeling approach (RM), have been proposed to account for the causal influence of BCS on MWT (Jamrozik et al. 2017; Ojo et al. 2025b). In contrast to phenotypic pre-adjustment, which operates on the observed phenotype, the RM operates on the underlying genetic effects contributing to trait expression. Specifically, the RM models the causal effect of BCS on MWT and partitions breeding values into direct genetic effects on MWT and indirect genetic effects mediated through association with BCS. Consequently, RM preserves the genetic architecture underlying the relationship between the traits while identifying the component of genetic variation in MWT that is independent of BCS (Valente et al. 2013). Therefore, the RM allows the evaluation of MWT that is genetically independent of BCS.

Although our previous study showed that model choice can impact sire rankings, resulting in more than 20% re-ranking among the top 10% of sires across models (Ojo et al. 2025b), the extent to which these differences translate to variation in genomic architecture remains unknown. To the best of our knowledge, no study has compared the genetic architecture of phenotypically pre-adjusted MWT (MWT_adj_) and of MWT genetically independent of BCS (MWT_RM_). This gap limits the understanding of whether these approaches identify the same biological pathways or lead to different conclusions about the loci underlying cow size. Therefore, the objective of this study was to determine whether phenotypic pre-adjustment and the RM capture different genetic architectures of MWT or merely produce statistical artifacts, and whether these alternative approaches influence the biological interpretation of the genetic basis underlying MWT in American Angus cattle. Therefore, the first objective of this study was to compare the effects of phenotypic pre-adjustment and the RM on the outcome of genome-wide association studies (GWAS), including the detection of significant SNPs, candidate genes, and enriched biological pathways. Secondly, we evaluated whether phenotypic pre-adjustment and RM revealed differences in the genetic architecture underlying MWT in American Angus cattle or merely produced statistical artifacts arising from model specification.

## Materials and methods

Phenotypic records, pedigree, and genomic information used in this study were provided by the AAA through Angus Genetics Inc.; therefore, no approval from an Animal Care and Use Committee was required. This study used the same datasets and quality control protocols reported in Ojo et al. (2025b). Briefly, the analysis included only Angus cows with recorded MWT and BCS. Cows younger than 1 yr and older than 15 yr were excluded from the dataset because few animals were represented at these ages. Contemporary groups (CG) were created by combining herd, year, and season of MWT measurement, and CG containing fewer than three animals were removed. Additionally, MWT observations that deviated by more than three standard deviations from the CG mean were excluded, and CG exhibiting no variation in BCS were also discarded. After quality control, 381,496 MWT and BCS records from 209,491 cows distributed across 12,765 CG were retained for analysis. The average (SD) number of animals per CG was 29.86 (50.63), the average (SD) number of records per CG was 29.89 (50.69), and the average number of MWT/BCS records per cow was 1.82. In addition to the raw phenotypes, single MWT_adj_ records were obtained for each of the 209, 491 cows. These records were pre-adjusted to a standard BCS of 6 and an age of 6 yr using the equations implemented in the official AAA genetic evaluation. Because these pre-adjusted records represent a single standardized measurement per animal, CG for them was defined solely by the combination of herd and year of birth.

### Genotypic information

A total of 45,606 cows were genotyped and imputed to a common marker density of 54,609 SNP. Imputation was performed by Angus Genetics Inc. using the FImpute software (Sargolzaei et al. 2014) as part of their official genomic evaluation pipeline. Among these, 33,764 animals had both genotypic and phenotypic information, whereas 11,842 were genotyped animals related to the animals with phenotypes. Genomic quality control (QC) was performed using the preGSf90 program (Aguilar et al. 2014). Quality control steps removed individuals and SNP with call rates below 0.90, markers located on non-autosomal chromosomes, SNP with unknown or duplicated genomic positions, and variants with a minor allele frequency (MAF) < 0.05. SNP showing an absolute difference > 0.15 (*P*-value ≥ 1 × 10^−6^) between observed and expected heterozygosity under Hardy–Weinberg equilibrium, as described by Wiggans et al. (2009), were also removed, as such deviations may indicate potential genotyping errors. After these, 51,410 SNP and 45,205 animals were available for further analyses. Due to a limitation on the version of the programs used in this study, a random sample of 20,000 genotyped animals with phenotypic records was selected for the GWAS.

### Single-step genome-wide association study using MWT_adj_

Single-step genome-wide association study (ssGWAS) was performed using the BLUPF90+ family of programs (Misztal et al. 2002, 2020; Lourenco et al. 2020), following the approach described by Wang et al. (2012). The mixed-model equation was solved using the following single-trait animal model:


(1) $$y^{*}=Xb^{*}+\mathrm{Za}+e$$

where y* is the vector of pre-adjusted observations (i.e. 209,491 MWT_adj_ provided by AAA); b* is the vector of fixed effects, including contemporary group (defined as the combination of herd-year of birth), and parity; **a** is the vector of random additive genetic effects; and **e** is the vector of random residual effects. The **X** and **Z** are the incidence matrices associating **b** and **a** to the phenotypic observations, respectively. All animals had a single MWT_adj_ record, and therefore, no permanent environmental effect was included in the model. This model assumes that **a**∼$N(0,H\sigma_{a}^{2}),$ and **e**∼$N(0,I\sigma_{e}^{2})$, where **H** is the combined pedigree-genomic relationship matrix as described in (Aguilar 2010), constructed using the preGSF90 program; **I** is an identity matrix; and $\sigma_{a}^{2}$ and $\sigma_{e}^{2}$ are the additive genetic and residual variances, respectively. The variance components were fixed to the pedigree-based estimates reported in Ojo et al. (2025b). These estimates were retained to maintain consistency with the analytical framework used in our previous GWAS of unadjusted MWT (Ojo et al. 2026), thereby allowing differences in GWAS results to be attributed primarily to differences in modeling approaches.

Estimates of SNP effects were derived by back-solving the genomic estimated breeding values (GEBV) predicted using equation 1. In this context, first, breeding values for genotyped animals were expressed as (Misztal et al. 2020):


(2) a=Zu

where **a** is the vector of GEBV for genotyped individuals calculated using BLUPF90+; **Z** is the incidence matrix relating individuals to SNP marker effects; and **u** is the vector of SNP substitution effects. The SNP effects were then estimated using the postGSF90 program (Aguilar et al. 2019) as:


(3) $$\hat{u}=Z^{\prime }{\left(\mathrm{ZI}Z^{\prime }\right)}^{-1}\hat{a}$$

where **û** is a vector of estimated SNP marker effects; **I** is an identity matrix; **Z** is the incidence matrix relating individuals to SNP marker effects; and $\hat{a}$ is the vector of GEBV. The SNP effects were assumed to follow the infinitesimal model, with equal variance across loci. Given the genetic homogeneity characteristic of the Angus breed (Mulim et al. 2025), consistent with the principal component analysis performed in our dataset (Figure S1, see online supplementary material for a color version of this figure), principal components were not included as covariates in the statistical models. Approximate *P*-values for each SNP were also obtained with the postGSF90 program (Aguilar et al. 2019) as:


(4) $$p_{i}=2(1-\Phi (|\frac{\alpha_{i}}{SD(\alpha_{I})}|))$$

where α_i_ is the SNP effect estimate; SD is the standard deviation; and Φ is the standard normal cumulative distribution function. To control for multiple testing, a Bonferroni correction (alpha = 0.05) was applied based on the number of independent chromosomal segments (Corbin et al. 2012). The number of segments was estimated using the average chromosome length and the effective population size (Ne = 182; Lozada-Soto et al. 2020), providing chromosome-wide significance thresholds (Goddard et al. 2011). The proportion of genetic variance explained by each chromosome was calculated following the approach described by Wang et al. (2012):


(5) $$\frac{\mathrm{Var}\;(a_{i})}{\sigma_{a}^{2}}\;\times 100\%=\frac{\mathrm{Var}\;(\sum_{j=1}z_{j}{\hat{u}}_{j})}{\sigma_{a}^{2}}\;\times 100\%$$

where $a_{i}$ is the genetic value for the SNP on the i^th^ chromosome; $\sigma_{a}^{2}$ is the total additive genetic variance; $z_{j}$ is the genotype vector (0, 1, 2) for the j^th^ SNP; and ${\hat{u}}_{j}$ is the estimated effect for the j^th^ SNP. The total variance explained by each chromosome was obtained by summing the contributions of all significant SNP located on that chromosome. These values were normalized to express each chromosome’s contribution as a percentage of the total variance explained by all SNP.

### ssGWAS using MWT_RM_

Unlike the ssGWAS for pre-adjusted MWT that uses MWT_adj,_ the RM has its parameters estimated from a multiple-trait model between MWT and BCS to account for the genetic correlation between them, as described by Jamrozik et al. (2017). The multiple-trait model used in this study is similar to the model defined in Ojo et al. (2025b), which can be defined as:


(6) $$y_{i}=X_{i}b^{*}+a_{i}^{*}+{\mathrm{pe}}_{i}^{*}+e_{i}^{*}$$

where $y_{i}$ is the vector of MWT and BCS records for animal i; $b^{*}$ is the vector of fixed effects, including CG (defined as the combination of herd-year-season of trait measurement), parity, age in years, and age in days fitted as a linear covariate nested within age in years; $X_{i}$ is the incidence matrix associating $b^{*}$ to $y_{i}$, $a_{i}^{*}$ is a vector of additive genetic effects; ${\mathrm{pe}}_{i}^{*}\;$is a vector of permanent environmental effects; and $e_{i}^{*}$ is a vector of residuals. Vectors $a_{i}^{*},\;{\mathrm{pe}}_{i}^{*},\;$and $e_{i}^{*}$ are assumed to follow a joint normal distribution with mean of 0 and var($a_{i}^{*}$) = $G^{*}=\;[g_{\mathrm{ij}}^{*}]$, var(${\mathrm{pe}}_{i}^{*}$) = $P^{*}$, and var($e_{i}^{*}$) = $R^{*}$. The model assumes that $a_{i}^{*}$ ∼ $N(0,H⨂G^{*}),\;{\mathrm{pe}}_{i}^{*}$ ∼ $N(0,H⨂P^{*})$, and $e_{i}^{*}$ ∼ $N(0,I⨂R^{*})$, where **H** and **I** are as described in equation 1.

To separate the genetic effect on MWT that is independent of BCS, we used the RM. Conceptually, the RM imposes a causal structure where BCS can influence MWT, but not vice-versa. Under this structure, the part of MWT that is genetically explained through BCS can be removed, leaving only the direct genetic effect on MWT. This causal relationship is represented by the matrix:


(7) $$\Lambda =[\begin{array}{cc} 1 & 0\\ -\lambda & 1 \end{array}].$$

The RM equivalent to equation 6 is defined as (Jamrozik and Miller 2014; Jamrozik et al. 2017):


(8) $${\Lambda y}_{i}=X_{i}b+a_{i}+{\mathrm{pe}}_{i}+e_{i},$$

where the recursive coefficient parameter $\lambda =g_{12}^{*}/g_{11}^{*}$ quantifies how much the genetics of BCS contributes to MWT. Using **Λ**, the solutions from the multiple-trait model can be transformed to obtain the RM parameterization (full equations are provided in Supplementary Note 1). The additive genetic effects for MWT can then be partitioned as follows:


(9) $$a_{i}^{\prime }={\left[a_{i1}^{*},a_{i2}^{*}-\lambda a_{i1}^{*}\right]}^{\prime }$$

where $a_{i2}^{*}-\lambda a_{i1}^{*}$is the direct genetic effect on MWT; and ${\lambda a_{i1}^{*}=a}_{i2}^{*}-(a_{i2}^{*}-\lambda a_{i1}^{*})$ represents the indirect genetic effect on MWT due to its correlation with BCS (indirect). After considering the relationship between MWT and BCS in the RM, we can define the direct MWT as:


(10) $$\mathrm{MWT}-\mathrm{direct}=\mathrm{MWT}-\lambda_{\mathrm{MWT}/\mathrm{BCS}}\times \mathrm{BCS}$$

where MWT-direct represents MWT genetically independent of BCS; and $\lambda_{\mathrm{MWT}/\mathrm{BCS}}$ is computed as the ratio of the genetic covariance between the two traits to the genetic variance of BCS. Thus, λ_MWT/BCS_*× BCS* represents the portion of MWT that is genetically mediated through BCS, and subtracting this component isolates the direct genetic effect on MWT. The variance components for the additive, permanent, and residual effects were fixed to the pedigree-based estimates reported in Ojo et al. (2025b). Estimates of SNP effects and proportion of variance explained by each SNP were computed as described earlier (equations 2–5).

### Comparison of modeling strategies in terms of SNP effects and GEBV

To evaluate the concordance between the different modeling strategies, a comparison between the results from the ssGWAS using MWT_adj_ and MWT_RM_ was performed by calculating the Pearson correlation coefficients for three distinct scenarios: 1) consistency of the estimated SNP effects across the entire genome, calculated as the Pearson correlation coefficient for all SNP across the genome as well as for the subset of SNP common to both modeling strategies; 2) consistency in the level of the animals’ overall genetic merit, calculated as the Pearson correlation coefficient calculated between the GEBV derived from each model; and 3) consistency in the identification of significant loci, based on the statistical evidence for association. In this last case, for the subset of SNP that were common to both modeling strategies, the –log10 transformed *P*-values were calculated. The Pearson correlation coefficient between these transformed *P*-values was then computed to evaluate the degree of agreement in the significance rankings of the shared SNP.

### Functional analyses and biological interpretation of ssGWAS results

Beyond statistical comparison, it was essential to determine whether the choice of modeling strategy (MWT_adj_ vs. MWT_RM_) influenced the biological interpretation of the results. Therefore, a functional analysis was performed to elucidate and compare the biological pathways and gene networks associated with the significant markers identified by each model. Gene annotations were performed using the GALLO package (Fonseca et al. 2020) available in the R software (R Core Team 2024), using the ARS-UCD2.0 bovine genome assembly (Rosen et al. 2020). A genomic window spanning 100 kb upstream and downstream of each significant SNP was used to identify nearby QTLs and genes. This threshold was defined based on the extent of linkage disequilibrium (LD) decay observed in the studied population, which has been previously reported in Ojo et al. (2026). Identified positional candidate genes were further analyzed using the R package gprofiler2 (Kolberg et al. 2020) to perform functional enrichment. The positional candidate genes were analyzed using functional enrichment of gene ontology (GO) terms for biological process (BP), molecular function (MF), and cellular component (CC), as well as the Human Phenotype Ontology (HP), and the Kyoto Encyclopedia of Genes and Genomes (KEGG) pathways.

Similarly, QTL annotation was performed using the same set of significant SNP and 100 kb window strategy, using the GALLO package (Fonseca et al. 2020) and the ARS-UCD2.0 genome assembly as reference (Rosen et al. 2020). Annotated regions were then compared against previously reported cattle QTL from the Animal QTL Database (Hu et al. 2013) to identify overlaps and assess potential biological relevance. Enrichment analysis was performed to test for over-representation of the identified QTL within major trait categories to allow for further exploration of the differences between phenotypic effects associated with related genomic regions from both models. The enriched QTL were defined based on a false discovery rate (FDR) < 0.05. The relationship between identified positional candidate genes and enriched QTL was further investigated using the NetVis function in GALLO, which represented genes and QTL as nodes and shared genomic associations as edges.

## Results

### ssGWAS for MWT_adj_ vs. MWT_RM_

**Figure 1:**
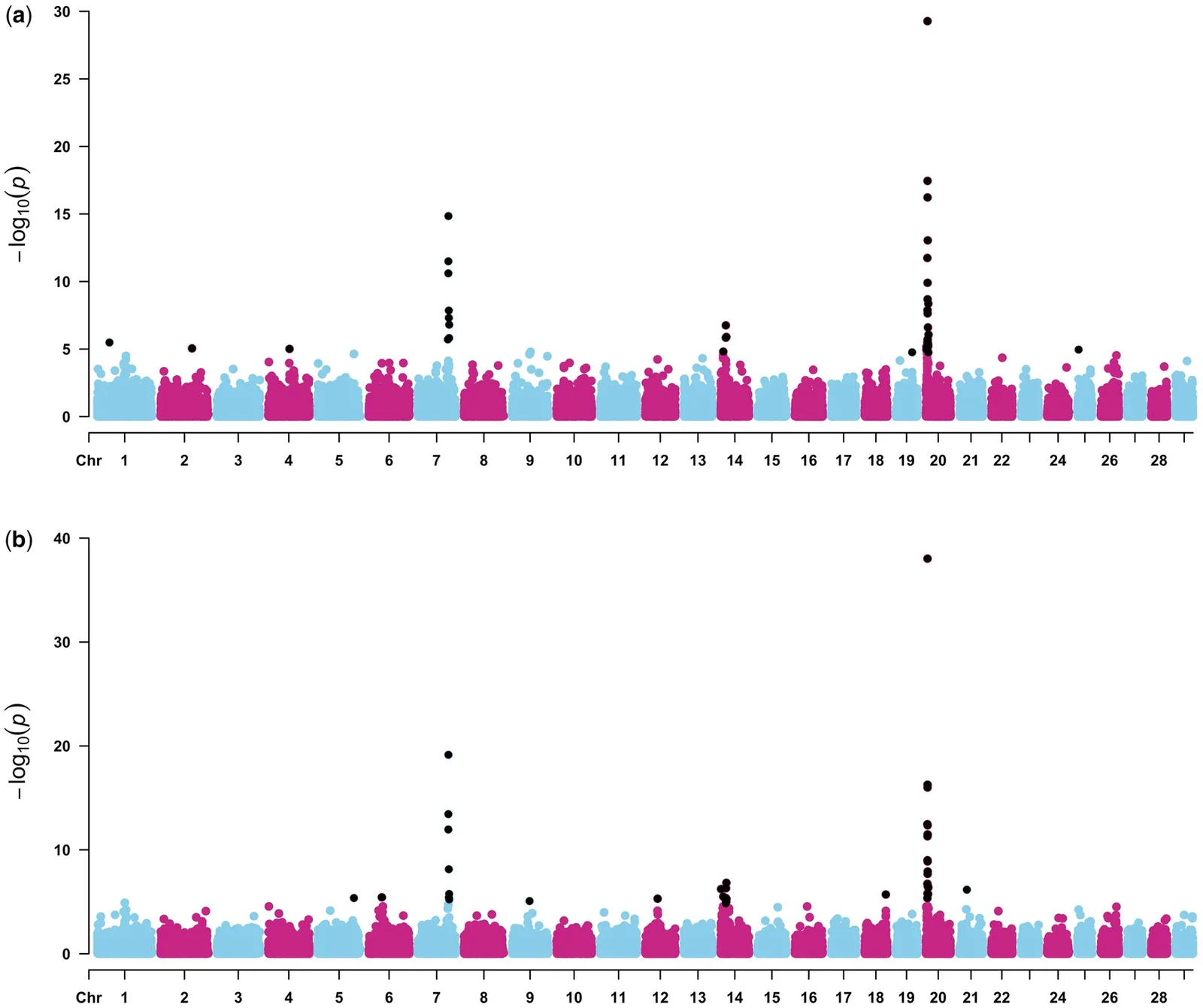
Manhattan plots based on–log_10_(p) values for a) phenotypically pre-adjusted mature cow weight, MWT_adj_; b) mature cow weight that is genetically independent of body condition score using the recursive approach, MWT_RM_; in American Angus cattle population. Black dots represent the significant markers.

**Figure 2:**
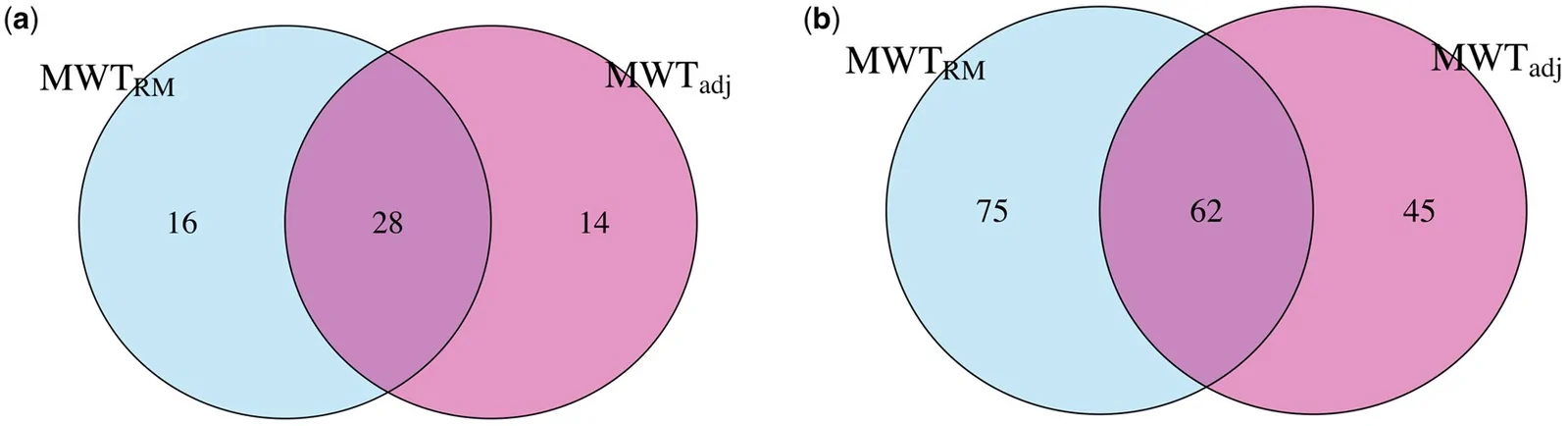
Venn diagram of the number of a) significant SNP and b) genes shared between phenotypically pre-adjusted mature cow weight, MWT_adj_; and mature cow weight that is genetically independent of body condition score using the recursive approach, MWT_RM_; in American Angus cattle population.

The estimated number of independent chromosomal segments (Me) per chromosome and the corresponding adjusted chromosome-wide significance thresholds are reported in Table S1. Figure 1 shows the Manhattan plot for MWT_adj_ and MWT_RM_ in the American Angus cattle population. A total of 42 and 44 significant SNP markers were identified for MWT_adj_ and MWT_RM_, respectively. Significant associations for MWT_adj_ were detected on eight chromosomes (i.e. BTA1, BTA2, BTA4, BTA7, BTA14, BTA19, BTA20, and BTA25), whereas significant associations for MWT_RM_ were identified on nine chromosomes (i.e. BTA5, BTA6, BTA7, BTA9, BTA12, BTA14, BTA18, BTA20, and BTA21). For both modeling approaches, the greatest concentration of significant SNPs was observed on BTA7, BTA14, and BTA20. For MWT_adj_, these chromosomes contained 8, 4, and 25 significant SNP, respectively, while for MWT_RM_ they contained 8, 9, and 21 significant SNP, respectively. Comparison of the two analyses revealed that 28 significant SNP were shared between the two approaches. Figure 2 shows the Venn diagrams illustrating the shared number of significant SNP and genes associated with MWT_adj_ and MWT_RM_.

The significant SNP explained 3.93% of the genetic variance of MWT_adj_, with those on BTA20, BTA7, and BTA14 accounting for 2.89%, 0.79%, and 0.20%, respectively. In the MWT_RM_, the total variance explained was 4.29%, with BTA20, BTA7, and BTA14 contributing 3.00%, 0.86%, and 0.36%, respectively. The Manhattan plot showing the percentage of variance explained by each SNP can be found in Figure S2 (see online supplementary material for a color version of this figure). In addition, the Pearson correlation coefficient between SNP effects on shared SNP was 0.76 and close to unity across all SNP. The correlation between GEBV derived from both modeling strategies was 0.87, whereas the correlation between –log_10_(p) values was 0.66 across all SNP and 0.95 for shared SNP.

A total of 107 genes were associated with MWT_adj_, including 47 protein-coding genes, 54 long non-coding RNA, 2 microRNA, 1 pseudogene, 1 ribosomal RNA, and 2 small nuclear RNA. For MWT_RM_, 137 genes were associated, comprising 69 protein-coding genes, 64 long non-coding RNA, 1 microRNA, 1 ribosomal RNA, and 2 small nuclear RNA. A total of 62 genes were common to both modeling strategies. Among the associated genes, 50 were functionally annotated for MWT_adj_, 70 for MWT_RM_, and 21 were shared between both modeling strategies. Table 1 shows the functionally annotated genes uniquely associated with MWT_adj_ and MWT_RM_, as well as those shared between both modeling strategies.

**Table 1:** Unique and shared functionally annotated genes associated with phenotypically pre-adjusted mature cow weight, MWT_adj_, and mature cow weight that is genetically independent of body condition score using the recursive approach, MWT_RM_.

Modeling strategy	Candidate genes
**MWT_adj_ only**	(BTA20)*ARHGEF28*, (BTA20)*FAM169A*, (BTA20)*NSA2*, (BTA20)*GFM2*, (BTA20)*HEXB*, (BTA20)*SPDL*, (BTA20)*DOCK2*, (BTA25)*ADCY9*, (BTA25)*SRL*, (BTA25)*TFAP4*, (BTA19)*DDX42*, (BTA19)*FTSJ3*, (BTA19)*PSMC5*, (BTA19)*SMARCD2*, (BTA19)*TCAM1*, (BTA19)*GH1*, (BTA19)*CD79B*, (BTA19)*SCN4A*, (BTA19)*PRR29*, (BTA19)*bta-mir-10173*, (BTA4)*DPY19L1*, (BTA4)*NPSR1*, (BTA2)*CYP20A1*, (BTA2)*ABI2*, (BTA2)*RAPH1*, (BTA1)*HTR1F*, (BTA1)*CGGBP1*, (BTA1)*ZNF654*, (BTA1)*U4*
**MWT_RM_ only**	(BTA14)*FER1L6*, (BTA14)*ZHX1*, (BTA14)*C14H8orf76*, (BTA14)*FAM83A*, (BTA14)*TBC1D31*, (BTA14)*ANXA13*, (BTA14)*MTSS1*, (BTA14)*FAM91A1*, (BTA14)*EPPK1*, (BTA14)*NRBP2*, (BTA14)*PUF60*, (BTA14)*SCRIB*, (BTA14)*IQANK1*, (BTA14)*FAM83H*, (BTA14)*ZNF623*, (BTA14)*MAPK15*, (BTA14)*CCDC166*, (BTA14)*GFUS*, (BTA12)*XPO4*, (BTA12)*EEF1AKMT1*, (BTA12)*IFT88*, (BTA12)*CRYL1*, (BTA18)*ZNF784*, (BTA18)*ZNF865*, (BTA18)*ZNF524*, (BTA18)*FIZ1*, (BTA18)*ZNF579*, (BTA18)*SBK3*, (BTA18)*SBK2*, (BTA18)*SSC5D*, (BTA18)*ZNF628*, (BTA18)*C19orf85*, (BTA18)*ISOC2*, (BTA18)*SHISA7*, (BTA18)*UBE2S*, (BTA18)*RPL28*, (BTA18)*TMEM238*, (BTA18)*TMEM190*, (BTA18)*IL11*, (BTA5)*FGF6*, (BTA5)*FGF23*, (BTA5)*TIGAR*, (BTA5)*CCND2*, (BTA21)*SLC28A1*, (BTA21)*PDE8A*, (BTA21)U5, (BTA21)*ALPK3*, (BTA9)*COQ3*, (BTA9)*FAXC*
**Shared annotated genes**	(BTA20)*CREBRF*, (BTA20)*BNIP1*, (BTA20)*NKX2-5*, (BTA20)*STC2*, (BTA20)*BOD1*, (BTA20)*ENC1*, (BTA20)*DUSP1*, (BTA20)*RPL26L1*, (BTA20)*ATP6V0E1*, (BTA20)*NEURL1B*, (BTA20)*SH3PXD2B*, (BTA20)*5S_rRNA*, (BTA20)*bta-mir-584-6*, (BTA20)*ERGIC1*, (BTA7)*ARRDC3*, (BTA14)*NT*, (BTA14)*ATAD2*, (BTA14)*TRIB1*, (BTA14)*FBXO32*, (BTA14)*NSMCE2*, (BTA18)*U6*

### Functional analyses and biological interpretation of ssGWAS results

**Figure 3:**
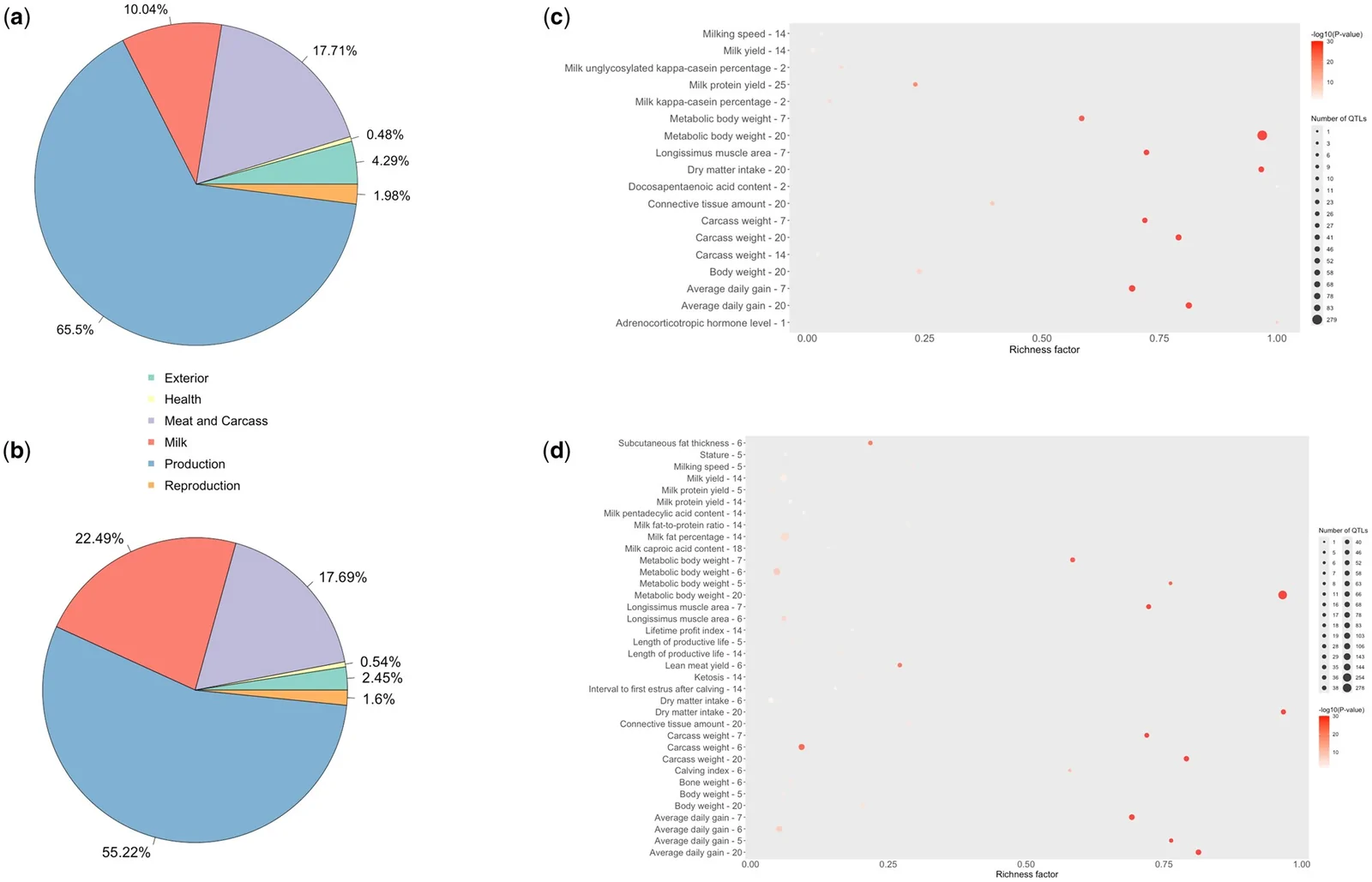
Chart of the trait-types associated with the quantitative trait loci (QTL) overlapping with the genomic regions associated with a) phenotypically pre-adjusted mature cow weight, MWT_adj_; b) mature cow weight that is genetically independent of body condition score using the recursive approach, MWT_RM_. QTL enrichment for the significant genomic regions associated with c) phenotypically pre-adjusted mature cow weight, MWT_adj_; d) mature cow weight that is genetically independent of body condition score using the recursive approach, MWT_RM_, in American Angus cattle population. The y-axis represents QTL categories, the x-axis shows the richness factor (calculated as the number of genes annotated in this study associated with a specific QTL divided by the total number of genes within that QTL), bubble area corresponds to the number of associated QTL, and bubble color indicates the *P*-value (darker color denotes higher significance).

Figure 3 shows the distribution of trait-types associated with QTL overlapping the significant genomic regions and the richness factor for enriched QTL associated with MWT_adj_ and MWT_RM_. Enrichment analysis identified 18 loci related to MWT_adj_. The enriched QTL included dry matter intake (BTA20), carcass weight (BTA7, BTA14, BTA20), metabolic body weight (BTA7, BTA20), average daily gain (BTA7, BTA20), and body weight (BTA20).

For MWT_RM_, the enrichment analysis identified 36 loci associated with multiple growth and production-related traits. These included average daily gain (BTA5, BTA6, BTA7, BTA20), body weight (BTA5, BTA20), bone weight (BTA6), calving index (BTA6), and carcass weight (BTA6, BTA7, BTA20). Across both modeling strategies, metabolic body weight, dry matter intake, average daily gain, *longissimus* muscle area, and carcass weight were among the trait categories with the highest enrichment significance (darker red circles), richness factors, and numbers of associated QTL. Furthermore, we identified 29 and 31 pathways related to MWT_adj_ and MWT_RM,_ respectively. No pathways based on gene ontology terms were common to both modeling strategies. The pathways associated with MWT_adj_ revealed significant terms primarily related to glycan degradation, glycosphingolipid biosynthesis, and glycosaminoglycan metabolism, as identified using KEGG and HP categories. For MWT_RM_, the pathway enrichment mapped almost exclusively to highly coordinated fibroblast growth factor receptor (FGFR1, FGFR2, FGFR4) signaling cascades, and phosphoinositide 3-kinase (PI3K)-dependent responses, and their regulatory mechanisms.

**Figure 4:**
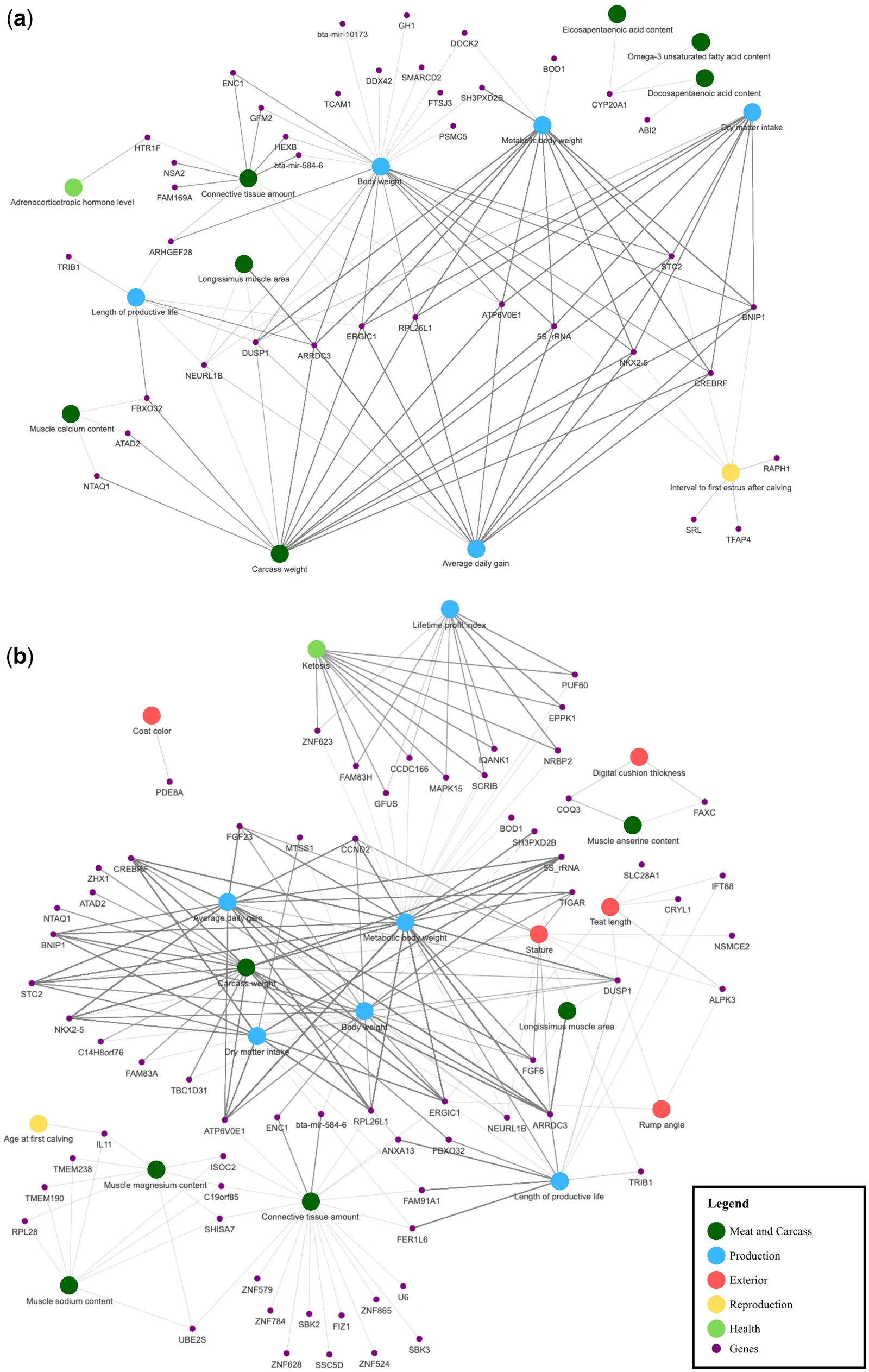
Interaction network composed of quantitative trait loci (QTL) terms (green) and genes (purple) associated with a) phenotypically pre-adjusted mature cow weight, MWT_adj_; b) mature cow weight that is genetically independent of body condition score using the recursive approach, MWT_RM_.

A gene–trait interaction network was constructed to visualize the relationships between positional candidate genes and associated QTL terms in MWT_adj_ and MWT_RM_ (Figure 4). For MWT_adj_, the network revealed multiple interconnected gene–QTL relationships, particularly within meat and carcass and production-related categories. Several trait categories were linked by two or more genes; for example, *NT*, *ATAD2*, and *FBXO32* connected muscle calcium content with carcass weight. The only isolated module in the MWT_adj_ network involved *CYP20A1* and *ABI2*, which were associated exclusively with three meat- and carcass-related QTL terms. For MWT_RM_, genes associated with one production-related trait were associated with other production traits or other trait categories. Isolated modules were also observed here, including connections between digital cushion thickness and muscle anserine content and *COQ3* and *FAXC* genes, and between coat color and the *PDE8A* gene. Carcass weight shared genes with average daily gain, metabolic body weight, body weight, and dry matter intake. In contrast to MWT_adj_, which did not identify any exterior-related QTL terms, MWT_RM_ analysis revealed five such terms, including stature, teat length, and rump angle. In both modeling strategies, one health-related and one reproduction-related QTL term were identified.

## Discussion

### Genomic regions associated with MWT_adj_ and MWT_RM_

The patterns of significant SNP across chromosomes highlight both consistencies and differences between modeling strategies. The concentration of significant SNP on BTA7, BTA14, and BTA20 under both MWT_adj_ and MWT_RM_ mirrors the results from the unadjusted MWT in a previous study (Ojo et al. 2026), suggesting that these chromosomes harbor key loci influencing MWT in Angus cattle (Figure 1). Notably, 27 significant SNP were shared between the unadjusted MWT from the previous study and the two modeling approaches evaluated here, highlighting the robustness of these core genomic signals despite differences in the treatment of BCS and the number of genotyped animals. The slightly lower number of SNP detected in MWT_adj_ is consistent with the expectation that phenotypic pre-adjustment for a correlated trait may reduce or alter the detection of some associations (Aschard et al. 2015; Melo et al. 2016). Likewise, the RM identified additional loci that may reflect the direct genetic component of MWT after accounting for its recursive relationship with BCS. Although this consistency supports the stability of the associations identified by the RM, it does not constitute independent validation that one modeling strategy more accurately reflects the true underlying genetic architecture of the trait. Overall, the consistent identification of the major genomic regions on BTA7, BTA14, and BTA20 across both approaches supports the robustness of these loci in the genetic architecture of MWT (Figures 1 and 3).

The variance explained by the significant SNP also illustrates the impact of both model choice and sample size on the genetic architecture captured (Purcell et al. 2003; Chitre et al. 2023). In the unadjusted MWT from a previous study, the total variance explained by significant SNP was 6.36%, with BTA20, BTA14, and BTA7 contributing 3.85%, 0.83%, and 1.38%, respectively (Ojo et al. 2026). In contrast, MWT_adj_ and MWT_RM_ captured total variances of 3.93% and 4.29%, respectively (Figure S2, see online supplementary material for a color version of this figure). Notably, MWT_RM_ captures slightly more genetic variance than MWT_adj_ and is closer to the variance observed in the unadjusted MWT. This likely reflects the fact that RM removes only the genetic dependence on BCS, preserving direct genetic effects on MWT, whereas phenotypic pre-adjustment reduces environmental noise but can inadvertently remove both environmental and some correlated genetic variance, thereby lowering the variance captured by significant SNP (Hartwig et al. 2021; Varona and González-Recio 2023).

The magnitude and direction of each variant’s contribution to the phenotype are quantified by SNP effects, whereas –log_10_(p) values primarily reflect the strength of statistical evidence for association, not effect size (Aguilar et al. 2019). Because significance is strongly influenced by factors such as sample size, allele frequency, and local linkage disequilibrium structure (Visscher et al. 2017), two models can show similar –log_10_(p) patterns, even when their underlying effect-size estimates differ. In our results, the correlation between –log_10_(p) values for shared significant SNP was high (0.95), indicating that both MWT_adj_ and MWT_RM_ identify similar genomic regions associated with MWT. However, when considering all SNP genome-wide, the –log_10_(p) correlation declined to 0.66, reflecting model-dependent differences in the distribution of weaker association signals, which is expected when phenotypes are pre-adjusted for a genetically correlated trait (Aschard et al. 2015).

The correlation of SNP effects across shared significant SNP remained moderate (0.76), indicating that although the same regions are detected as significant, the magnitudes of estimated effects differ slightly between MWT_adj_ and MWT_RM_. At the same time, the near-unity correlation of SNP effects when considering all suggests that differences concentrate around a subset of loci whose effects are entangled with body condition. Finally, the high GEBV correlation (0.87) shows that genomic prediction is robust to these model differences, consistent with previous reports that breeding value rankings often remain stable across reasonable alternative modeling strategies (Hayes et al. 2009). It is important to note, however, that these correlations can differ across subsets of the population; for example, the correlation between estimated breeding values for the top 10% bulls was lower (0.80; Ojo et al. 2025b).

### Biological pathways identified across MWT_adj_ and MWT_RM_

The functional annotation of genes associated with MWT_adj_ highlighted enrichment for pathways involved in glycosphingolipid and glycan metabolism, as evidenced by multiple HP ontology terms, including Abnormal glycosphingolipid metabolism, other glycan degradation, and glycosphingolipid biosynthesis. Although these pathways are defined from human clinical phenotypes, they reflect conserved metabolic processes in mammals (Seo and Lewin 2009). Glycosphingolipid and glycan metabolic pathways play central roles in cellular signaling, membrane structure, and energy metabolism (Schnaar 2004; Sandhoff and Sandhoff 2018), all of which can influence growth and body composition in mammals, including cattle (Hocquette et al. 2010). The identification of HP terms such as oligosacchariduria and increased urinary N-acetylglucosamine-rich oligosaccharide levels further supports a role for glycan and glycolipid processing in variation in mature weight (Wang et al. 2025). Muscle- and neuromuscular-related HP terms such as upper limb muscle weakness, proximal muscle weakness in lower limbs, muscle spasm, and limb joint contracture suggest that variations in genes affecting neuromuscular development or function may indirectly influence body weight by altering muscle deposition, mobility, or growth trajectories (Schiaffino and Reggiani 2011). Notably, none of these pathways overlapped with those identified for MWT_RM_ or unadjusted MWT, suggesting that phenotypic pre-adjustment resulted in a distinct pathway enrichment profile. Given the sensitivity of pathway enrichment analyses to the set of input genes and analytical parameters, these differences should be interpreted as differences in enrichment results rather than definitive evidence of distinct biological mechanisms.

Of the 31 pathways identified for MWT_RM_, 28 overlapped with the unadjusted MWT, while three Insulin-like Growth Factor 1 receptor (IGF1R)-related pathways were uniquely captured by the RM, including the IRS-related events triggered by IGF1R, IGF1R signaling cascade, and Signaling by Type 1 IGF1R. The IGF1R axis is a well-established regulator of postnatal growth, muscle development, and anabolic metabolism (Ma et al. 2019; Li et al. 2024). Its identification in MWT_RM_, but not MWT_adj_, is consistent with the possibility that the RM captures additional growth-related signaling pathways, although further validation would be required to confirm this interpretation. The shared pathways included insulin receptor signaling and IRS-mediated phosphoinositide 3-kinase cascades, which regulate glucose uptake, protein synthesis, and lipid metabolism (Saltiel and Kahn 2001; Copps and White 2012; Boucher et al. 2014), as well as FGFR signaling essential for skeletal growth, muscle differentiation, and adipose regulation (Shaul and Seger 2007; Vanhaesebroeck et al. 2012; Ornitz and Itoh 2015). The greater overlap between MWT_RM_ and the unadjusted MWT analysis is consistent with the expectation that the RM retains genetic variation associated with MWT while accounting for its recursive relationship with BCS. However, because pathway enrichment results are influenced by the underlying gene sets and analytical procedures, this concordance should be interpreted as evidence of greater similarity in enrichment profiles rather than definitive confirmation that one modeling strategy more accurately represents the underlying biological mechanisms impacting a trait.

### Positional candidate genes identified exclusively for MWT_adj_

Several candidate genes specific to MWT_adj_ have known functions in growth, skeletal development, feed efficiency, and carcass traits (Table 1; Figure 4a). For instance, the *GH1* gene was identified in the MWT_adj_ and has been associated with growth (Taylor et al. 1998), post-weaning average daily gain on feed in Angus cattle (Kneeland et al. 2004), average daily gain (ADG) in Nellore bulls (Borges et al. 2025), and carcass composition in beef cattle (Curi et al. 2006, 2010), including carcass and meat quality traits. Its detection in MWT_adj_ likely reflects both its direct influence on growth and pleiotropic effects on body composition. Other genes identified in MWT_adj_, such as *RAPH1*, *CD79B*, and *SCN4A*, have also been linked to growth. The *CD79B* and *SCN4A* genes are associated with ADG in beef steers, while *RAPH1* is involved in cell migration, a function suggested to influence nutrient absorption by the rumen epithelium in beef steers (Li et al. 2019). In addition to *SCN4A* and *CD79B*, the *DDX42, FTSJ3*, and *SMARCD2* genes have been associated with intramuscular fat and fatty acid composition of the *Longissimus thoracis et lumborum* muscle in rabbits (Qiu et al. 2024), as well as forelimb and rear limb cannon bone circumference and rear limb metatarsal area bone mineral density in pigs (El Nagar et al. 2023). These associated genes show that MWT_adj_ captures loci with broader effects on muscling, skeletal structure, and body composition, likely reflecting both direct and indirect influences on MWT.

Additionally, the *FAM169A* and *GFM2* genes have been linked with carcass weight in Angus cattle (Baneh et al. 2025; Suarez et al. 2025). Like the *GFM2* gene, *NSA2* has been associated with reproductive traits, including retained placenta (Guarini et al. 2019), heifer embryonic loss (Galliou et al. 2020), spontaneous abortion (Suarez et al. 2025), gene expression in the cattle placenta (Davenport et al. 2024), and primiparous embryonic loss in dairy cows (Kiser et al. 2019), while *HEXB* has been linked with sire fertility traits in dairy cattle (Chen et al. 2022). The detection of several genes associated with fat deposition in the analysis of MWT_adj_ reflects that phenotypic pre-adjustment enables the identification of loci with direct effects on MWT and growth-related traits, while also revealing genes linked to adiposity. Interestingly, none of the 29 genes unique to MWT_adj_ had been previously detected in the unadjusted MWT analysis, indicating that phenotypic pre-adjustment introduces a distinct set of signals not present in the raw phenotype. In contrast, of the 21 functionally annotated genes shared between MWT_adj_ and MWT_RM_, 19 were also shared with the unadjusted MWT, highlighting that some biologically relevant loci recovered by the pre-adjustment overlap with the raw phenotype, even though the pre-adjustment also introduces unique, non-overlapping signals.

### Positional candidate genes identified exclusively for MWT_RM_

Genes uniquely captured by the MWT_RM_ are associated with growth, carcass composition, feed efficiency, and reproductive traits, perhaps highlighting the model’s power to identify loci with direct effects on mature weight beyond BCS (Table 1;  Figure 4b). For instance, *FER1L6* has been associated with yearling weight, carcass weight, and bone weight (Naserkheil et al. 2020; Niu et al. 2021), while *ZHX1* has been linked to carcass weight (Naserkheil et al. 2020), metabolic body weight (Hardie et al. 2017), and beef tenderness (Zhao et al. 2012), suggesting these loci influence both skeletal growth and meat quality. Other genes, including *CR4H8orF76*, *FAM83A*, *TBC1D31*, and *ANXA13*, are similarly associated with carcass weight (Naserkheil et al. 2020; Srikanth et al. 2020; Lee et al. 2023), with *ANXA13* also linked to yearling weight (Naserkheil et al. 2020, 2022) and pathways related to beef marbling score (Lim et al. 2014). The *CR4H8orF76* gene is also associated with scrotal circumference in Nellore bulls (Irano et al. 2016), while the *ANXA13* is involved in calcium-dependent phospholipid binding and cellular growth regulation reflecting its potential role in muscle development and adipose deposition (Lim et al. 2014; Naserkheil et al. 2020).

Additional loci such as *MTSS1* and *FAM91A1* have been associated with carcass weight, yearling weight, and dry matter intake (Seabury et al. 2017; Naserkheil et al. 2020), suggesting pleiotropic effects on growth efficiency and feed utilization. Genes involved in reproductive performance, including *SBK2*, *ISOC2*, and *SHISA7*, are linked to cow and heifer fertility traits (e.g. age at first service, first service to conception, calving ease, calf survival, and daughter fertility), indicating that MWT_RM_ can capture loci that contribute to MWT through long-term reproductive and metabolic pathways (Yu et al. 2019; Chen et al. 2022). Other notable genes associated with MWT_RM_ include *IFT88*, which interacts with the testes-enriched gene *CFAP58*, which influences sperm motility in humans, cattle, and mice (Wei et al. 2024) and *UBE2S*, associated with tenderness and carcass traits (Rezende et al. 2021). Genes affecting skeletal and muscle morphology, such as *TMEM238* (productive lifespan) in U.S. Katahdin Sheep (Pinto et al. 2025) and *ALPK3* (loin eye area muscle) in Korean Native cattle and pigs (Kee et al. 2008; Kim et al. 2020). Finally, *SLC28A1*, a sodium-coupled nucleoside transporter highly expressed in the duodenum, is involved in pyrimidine absorption and shows correlated expression with *SLC28A2/28A3*, indicating potential effects on nutrient uptake and growth efficiency (Liao et al. 2008). The GWAS of MWT_RM_ detected loci with direct impact on MWT and related traits, including growth, skeletal development, feed efficiency, carcass composition, and reproduction. The diverse functions of the identified genes reflect the complex and pleiotropic genetic architecture underlying MWT in Angus cattle.

As stated earlier, all genes shared between the unadjusted MWT in a previous study and MWT_adj_ were also identified by the RM. Aside from the 19 functionally annotated genes shared among the unadjusted MWT, MWT_adj_, and MWT_RM_, MWT_RM_ shared 14 extra genes with MWT, indicating a closer alignment with the unadjusted biological architecture while still controlling for BCS at the modeling stage. However, these 14 additional genes, which were not detected in the pre-adjusted analysis, are biologically consistent with MWT. Some of the genes associated with MWT_RM_ but not MWT_adj_ include *FGF6*, *FGF23*, *TIGAR*, *CCND2*, *GFUS*, *MAPK15*, EPPK1, *NRBP2*, *PUF60*, and *SCRIB*, suggesting that through the RM growth and development-related loci whose signals were dampened or removed during phenotypic pre-adjustment, can be recovered. The *FGF6* plays a significant role in regulating muscle mass (Seabury et al. 2017), growth (Igoshin et al. 2019), body size (Ghoreishifar et al. 2020), stature and meat quality traits (Bolormaa et al. 2014), inherently linked to MWT. The *TIGAR* gene, which is also associated with stature, functions alongside *CCND2* to regulate growth-related metabolic pathways (Ghoreishifar et al. 2020). In addition, the *CCND2* gene is associated with ADG and metabolic body weight (Zhang et al. 2020), and notably, it is recognized as the second most significant gene associated with stature across mammals (Bolormaa et al. 2014), highlighting its relevance to skeletal growth and mature size. The recovery of these genes in MWT_RM_ but not in MWT_adj_ highlights how phenotypic pre-adjustment can mask biologically meaningful variation, whereas the RM retains the capacity to detect such loci.

### Shared genes between modeling strategies and implications for MWT biology

Among the loci identified by both MWT_adj_ and MWT_RM_, 21 genes were functionally annotated, representing a core set of genomic regions consistently associated with MWT across modeling strategies (Table 1). Of these 21 genes, 19 were previously associated with unadjusted MWT, and they exhibit pleiotropic effects across the three traits, with some affecting all three (MWT, mature cow height, and BCS) and others affecting only pairs of traits (Ojo et al. 2026). However, they all have known roles in growth, metabolism, and cellular regulation in mammals. For example, the *BOD1* gene has been associated with carcass weight in Angus cattle (Baneh et al. 2025) and with residual feed intake (De Las Heras-Saldana et al. 2019), suggesting a role in growth efficiency and tissue accretion. Genes such as *TRIB1* and *NSMCE2* were uniquely associated with unadjusted MWT in Angus cattle (Ojo et al. 2026). The *TRIB1* gene has been linked to mastitis and is involved in fat deposition in the longissimus dorsi muscle of heat-stressed Jinjiang cattle (Ibeagha-Awemu et al. 2025; Liang et al. 2025), while *NSMCE2* has been associated with carcass weight and yearling weight in Hanwoo cattle (Naserkheil et al. 2020). Genes linked with MWT and BCS, such as *ARRDC3*, have been linked to carcass traits in cattle, including hot carcass weight, rib-eye area, and marbling score (Wang et al. 2020; Baneh et al. 2025), as well as to measures of feed efficiency and growth, such as residual feed intake, dry matter intake, average daily gain, and metabolic body weight (Zhang et al. 2020). Studies in mice and human males indicate that *ARRDC3* plays a role in regulating obesity, indicating its involvement in energy balance and adiposity, which are key determinants of overall body size in cattle (Patwari et al. 2011; Patwari and Lee 2012). In addition, the *ENC1* gene has been associated with residual feed intake and carcass weight in Angus cattle (Zhang et al. 2020; Baneh et al. 2025), emphasizing its role in modulating feed efficiency and tissue growth, which are closely linked to body size and structural development.

Furthermore, both models identified genes such as *NEURL1B* and *SH3PXD2B*, which are associated with MWT and mature cow height in Angus cattle (Ojo et al. 2026). The *NEURL1B* gene has been shown to affect the longissimus dorsi muscle area, implicating it in skeletal muscle development (Li et al. 2017), while *SH3PXD2B* is involved in postnatal growth and tissue development, highlighting its significance in early life growth regulation (Mao et al. 2009). The comparison between MWT_adj_ and MWT_RM_ indicates that both models consistently capture a core set of genes strongly associated with MWT, demonstrating that these loci are robust to whether body condition is adjusted phenotypically or modeled as a covariate.

### Limitations and future directions

While this study provides important insights about the genetic architecture of MWT_adj_ and MWT_RM,_ several limitations should be acknowledged, and future research directions proposed. First, the number of records differed between the two modeling strategies because the RM incorporated repeated MWT and BCS measurements (mean = 1.82 records per animal), whereas MWT_adj_ relied on a single adjusted phenotype per animal. Although comparing these alternative modeling approaches was a primary objective of this study, it is difficult to quantify the extent to which the genetic and environmental relationships between MWT and BCS are retained or not in MWT_adj_. The RM uses repeated MWT and BCS observations and directly models the relationships between these traits. Consequently, differences observed between the two approaches may reflect not only differences in model specification but also differences in the information available to characterize the underlying genetic architecture, which may have influenced the detection of genetic associations.

Another limitation of this study is the use of a randomly selected subset of approximately 20,000 genotyped animals with phenotypic records for the GWAS. Although restricting the analysis to a subset reduces the total number of genotyped observations and may limit power to detect small-effect loci, the random sampling approach minimizes selection bias and maintains a representative sample of the genotyped population. Furthermore, the same subset of genotyped animals was used across both modeling strategies, ensuring that comparisons were not confounded by differences in sample composition. While the reduced sample size may have influenced the detection of marginal associations, we do not expect it to introduce systematic bias into the comparative assessment of the different approaches. Importantly, the GEBV were obtained using ssGBLUP, which incorporates pedigree relationships from both genotyped and non-genotyped animals, allowing information from the entire population to contribute to the genetic evaluation.

Future research should expand these findings by evaluating different populations, including those differing in breed composition, as well as exploring alternative sets of traits to assess the consistency of genomic signals across modeling strategies. This framework should also be applied to traits that vary widely in their genetic correlations. Assessing models across pairs of traits with low, moderate, and high correlations will help determine whether the strength of the underlying genetic relationship influences the degree to which recursive versus pre-adjusted approaches differ in the loci they detect. In addition, studies based on simulated data, where the underlying genetic architecture and causal variants are known, would provide a controlled framework for directly comparing these approaches and better understanding the conditions under which their performance diverges. Such comparisons would clarify whether the stability of genomic regions observed here is unique to mature cow size or a more general outcome of modeling moderately correlated biological traits. Additionally, the recently proposed Mendelian residual framework represents a promising future direction for studies specifically aimed at causal variant discovery, as it enables orthogonal testing of genetic effects while accounting for population structure (Cantet and Jensen 2025). By isolating Mendelian sampling effects from population structure, this approach may complement either phenotypic pre-adjustment or the RM strategies by improving the identification of causal variants that directly influence the trait.

## Conclusion

This study demonstrates that different treatments of BCS in the genetic evaluation of MWT, either through phenotypic pre-adjustment or recursive modeling, captured a shared core set of functionally important genes and genomic regions associated with growth, metabolism, and body composition. While MWT_adj_ can reduce environmental variation and reveal loci influenced by body condition, it may mask some minor-effect loci. Yet, it also introduces unique signals not detected in unadjusted MWT or MWT_RM_. In contrast, MWT_RM_ preserves direct genetic effects on MWT and recovers additional loci involved in growth, skeletal development, feed efficiency, and carcass traits. Both approaches identify pleiotropic genes affecting MWT, mature height, and BCS, highlighting the interconnected genetic architecture of growth traits. Overall, these results indicate that although the modeling strategy affects the detection of some loci and leads to divergence in enriched biological pathways, the underlying genetic architecture of MWT is largely consistent.

## Supplementary Material

skag231_Supplementary_Data

## Abbreviations

AAA: American Angus Association
BCS: body condition score
BP: biological processes
BTA: *Bos taurus* autosome
CC: cellular component
CG: contemporary groups
FDR: false discovery rate
GEBV: genomic estimated breeding values
GO: gene ontology
GWAS: genome-wide association studies
HP: human phenotype ontology
KEGG: Kyoto Encyclopedia of Genes and Genomes
MAF: minor allele frequency
MF: molecular function
MWT: mature cow weight
MWT_adj_: phenotypically pre-adjusted mature cow weight
MWT_RM_: mature cow weight genetically independent of BCS, obtained using the recursive approach
QC: quality control
QTL: quantitative trait loci
RM: recursive modeling approach
RNA: ribonucleic acid
SNP: single nucleotide polymorphisms
ssGWAS: single-step genome-wide association study
