# Supplementary material for: Disentangling mature cow weight and body condition score: a comparative single-step GWAS of phenotypic pre-adjustment vs. recursive modeling
Source: J Anim Sci. 2026 Jul 28;104:skag231. doi: 10.1093/jas/skag231 (PMC13600373; doi:10.1093/jas/skag231)
Supplement: Supplementary file 1 [file skag231_supplementary_data.docx]

**SUPPLEMENTARY MATERIAL**

**Supplemental figure 1.** Principal component analysis of the studied population showing the first two principal components (PC1 and PC2).

**Supplemental note 1.** Transformations Between Multivariate and Recursive Mixed Models

The MTM used in this study is defined as:

$\mathbf{y}_{\mathbf{i}} \mathbf{=}\mathbf{X}_{\mathbf{i}}\mathbf{b}^{\mathbf{*}}\mathbf{+}\mathbf{a}_{\mathbf{i}}^{\mathbf{*}}\mathbf{+}\mathbf{pe}_{\mathbf{i}}^{\mathbf{*}}\mathbf{+}\mathbf{e}_{\mathbf{i}}^{\mathbf{*}}$	[1]

We assumed further that $\boldsymbol{\Lambda=}\left[ \begin{matrix} 1 & 0 \\ -\lambda& 1 \end{matrix} \right]$ defines the causal structure between MWT and BCS. The RM equivalent to [1] is defined as:

$\boldsymbol{\Lambda y}_{\mathbf{i}} \mathbf{=}\mathbf{X}_{\mathbf{i}}\mathbf{b +}\mathbf{a}_{\mathbf{i}}\mathbf{+}\mathbf{pe}_{\mathbf{i}}\mathbf{+}\mathbf{e}_{\mathbf{i}}$**_,_**	[2]

where the RM can be reparametrized as a standard mixed model (MTM) by multiplying all terms of [2] by $\boldsymbol{\Lambda}^{\mathbf{-1}}$ as follows:

var($\mathbf{a}_{\mathbf{i}}$) = **G**, var($\mathbf{pe}_{\mathbf{i}}$) = **P**, var($\mathbf{e}_{\mathbf{i}}$) = **R** (diagonal matrix), and

$\mathbf{G}^{\mathbf{*}}\mathbf{=}\boldsymbol{\Lambda}^{\mathbf{-1}}\mathbf{G}\boldsymbol{\Lambda}^{\mathbf{'-1}}$,	$\mathbf{P}^{\mathbf{*}}\boldsymbol{=}\boldsymbol{\Lambda}^{\mathbf{-1}}\mathbf{P}\boldsymbol{\Lambda}^{\mathbf{'-1}}$,	$\mathbf{R}^{\boldsymbol{*}}\boldsymbol{=}\boldsymbol{\Lambda}^{\mathbf{-1}}\mathbf{R}\boldsymbol{\Lambda}^{\mathbf{'-1}}$,	[3]

$\mathbf{b}^{\mathbf{*}}\boldsymbol{=}\boldsymbol{\Lambda}^{\mathbf{-}\mathbf{1}}\mathbf{b}$,	$\mathbf{a}_{\mathbf{i}}^{\mathbf{*}}\boldsymbol{=}\boldsymbol{\Lambda}^{\mathbf{-}\mathbf{1}}\mathbf{a}_{\mathbf{i}}$,	$\mathbf{pe}_{\mathbf{i}}^{\mathbf{*}}\boldsymbol{=}\boldsymbol{\Lambda}^{\mathbf{-}\mathbf{1}}\mathbf{pe}_{\mathbf{i}}$ ,	$\mathbf{e}_{\mathbf{i}}^{\mathbf{*}}\boldsymbol{=}\boldsymbol{\Lambda}^{\mathbf{-}\mathbf{1}}\mathbf{e}_{\mathbf{i}}$

After getting the solutions for MTM [1], parameters of the RM [2] were derived using the recursive coefficient parameter $\lambda=g_{12}^{*}/g_{11}^{*}$ and relationships in [3].

Here, $\mathbf{y}_{\mathbf{i}}$ is the vector of MWT and BCS records for animal i, $\mathbf{b}^{\mathbf{*}}$ is the vector of fixed effects, $\mathbf{X}_{\mathbf{i}}$ is the incidence matrix associating $\mathbf{b}^{\mathbf{*}}$ to $\mathbf{y}_{\mathbf{i}}$, $\mathbf{a}_{\mathbf{i}}^{\mathbf{*}}$ is a vector of additive genetic effects, $\mathbf{pe}_{\mathbf{i}}^{\mathbf{*}}$is a vector of permanent environmental effects, $\mathbf{e}_{\mathbf{i}}^{\mathbf{*}}$ is a vector of residuals. Vectors $\mathbf{a}_{\mathbf{i}}^{\mathbf{*}}$**,** $\mathbf{pe}_{\mathbf{i}}^{\mathbf{*}}$ and $\mathbf{e}_{\mathbf{i}}^{\mathbf{*}}$ are assumed to follow a joint normal distribution with mean of 0 and var($\mathbf{a}_{\mathbf{i}}^{\mathbf{*}}$) = $\mathbf{G}^{\mathbf{*}}\boldsymbol{=}[g_{\mathrm{ij}}^{*}]$ **,** var($\mathbf{pe}_{\mathbf{i}}^{\mathbf{*}}$) = $\mathbf{P}^{\mathbf{*}}$, and var($\mathbf{e}_{\mathbf{i}}^{\mathbf{*}}$) = $\mathbf{R}^{\mathbf{*}}$. The model assumes that $\mathbf{a}_{\mathbf{i}}^{\mathbf{*}}$ ~ $N\left( 0,\mathbf{H}\bigotimes\mathbf{G}^{\mathbf{*}} \right),$ $\mathbf{pe}_{\mathbf{i}}^{\mathbf{*}}$ ~ $N\left( 0,\mathbf{H}\bigotimes\mathbf{P}^{\mathbf{*}} \right)$, and

$\mathbf{e}_{\mathbf{i}}^{\mathbf{*}}$ ~ $N(0,\mathbf{I}\bigotimes\mathbf{R}^{\mathbf{*}})$.

**Supplemental table 1.** Estimated effective number of independent chromosomal segments (Me), chromosome length (Mbp; megabase pairs), and adjusted chromosome-wide significance thresholds (threshold) for the 29 *Bos* *taurus* autosomes.

| **Chr** | **Length (Mbp)** | **Me*** | **Threshold** |
| --- | --- | --- | --- |
| 1 | 160.25 | 5,673.66 | 4.96 |
| 2 | 138.85 | 4,985.49 | 4.82 |
| 3 | 125.43 | 4,549.25 | 4.56 |
| 4 | 119.65 | 4,360.26 | 4.73 |
| 5 | 124.56 | 4,520.98 | 4.62 |
| 6 | 117.60 | 4,292.80 | 4.79 |
| 7 | 110.14 | 4,047.31 | 4.55 |
| 8 | 115.99 | 4,239.96 | 4.62 |
| 9 | 104.22 | 3,851.04 | 4.74 |
| 10 | 103.11 | 3,814.23 | 5.00 |
| 11 | 106.81 | 3,937.07 | 4.94 |
| 12 | 86.86 | 3,270.06 | 4.96 |
| 13 | 82.94 | 3,137.65 | 4.93 |
| 14 | 81.98 | 3,105.15 | 5.05 |
| 15 | 84.22 | 3,181.03 | 4.91 |
| 16 | 80.57 | 3,057.15 | 4.89 |
| 17 | 72.92 | 2,795.88 | 4.93 |
| 18 | 65.47 | 2,538.98 | 4.88 |
| 19 | 62.98 | 2,452.67 | 4.90 |
| 20 | 71.88 | 2,760.10 | 4.88 |
| 21 | 69.02 | 2,661.78 | 4.80 |
| 22 | 60.53 | 2,367.37 | 4.79 |
| 23 | 52.28 | 2,077.52 | 4.69 |
| 24 | 63.37 | 2,466.07 | 4.71 |
| 25 | 43.31 | 1,757.12 | 4.75 |
| 26 | 51.89 | 2,063.57 | 4.69 |
| 27 | 45.31 | 1,829.03 | 4.57 |
| 28 | 45.59 | 1,838.96 | 4.61 |
| 29 | 50.82 | 2,025.64 | 4.68 |

*****Me was calculated as (Me = $\frac{2\times Ne\times L}{\log(Ne\times L)})$, where Ne is the effective population size (182; Lozada-Soto et al., 2020), and L is the chromosome length in megabase pairs (Mbp). Chromosome-wide significance thresholds were computed as ($threshold= -\log_{10} \frac{0.05}{Me}$). Chr: chromosome**.**

(a)

(b)

**Supplemental figure 2.** Manhattan plots based on the proportion of additive genetic variance explained by each SNP for (a) phenotypically pre-adjusted mature cow weight, MWT_adj_; (b) mature cow weight that is genetically independent of body condition score using the recursive approach, MWT_RM_, in the American Angus Cattle population. Black dots represent the significant markers.
